# Shared Genetic Factors of Anxiety and Depression Symptoms in a Brazilian Family-Based Cohort, the Baependi Heart Study

**DOI:** 10.1371/journal.pone.0144255

**Published:** 2015-12-09

**Authors:** Tâmara P. Taporoski, André B. Negrão, Andréa R. V. R. Horimoto, Nubia E. Duarte, Rafael O. Alvim, Camila M. de Oliveira, José E. Krieger, Malcolm von Schantz, Homero Vallada, Alexandre C. Pereira

**Affiliations:** 1 Department and Institute of Psychiatry (LIM 23), University of São Paulo Medical School, São Paulo, SP, Brazil; 2 Laboratory of Genetics and Molecular Cardiology, Heart Institute, University of São Paulo Medical School, São Paulo, SP, Brazil; 3 Department of Physiology, Federal University of Juiz de Fora, Juiz de Fora, MG, Brazil; 4 Department of Biochemistry and Physiology, Faculty of Health and Medical Sciences, University of Surrey, Guildford, Surrey, United Kingdom; University of Wuerzburg, GERMANY

## Abstract

To investigate the phenotypic and genetic overlap between anxiety and depression symptoms in an admixed population from extended family pedigrees. Participants (n = 1,375) were recruited from a cohort of 93 families (mean age±SD 42±16.3, 57% female) in the rural town of Baependi, Brazil. The Hospital Anxiety and Depression Scale (HADS) was used to assess depression and anxiety symptoms. Heritability estimates were obtained by an adjusted variance component model. Bivariate analyses were performed to obtain the partition of the covariance of anxiety and depression into genetic and environmental components, and to calculate the genetic contribution modulating both sets of symptoms. Anxiety and depression scores were 7.49±4.01 and 5.70±3.82, respectively. Mean scores were affected by age and were significantly higher in women. Heritability for depression and anxiety, corrected for age and sex, were 0.30 and 0.32, respectively. Significant genetic correlations (ρ_g_ = 0.81) were found between anxiety and depression scores; thus, nearly 66% of the total genetic variance in one set of symptoms was shared with the other set. Our results provided strong evidence for a genetic overlap between anxiety and depression symptoms, which has relevance for our understanding of the biological basis of these constructs and could be exploited in genome-wide association studies.

## Introduction

The National Comorbidity Study in the US estimated that depressive disorder has a lifetime prevalence of approximately 16%, making it one of the most frequently diagnosed psychiatric disorders [1]. By 2020, major depressive disorder is projected to be the second leading cause of disability worldwide [2]. Anxiety disorders have a lifetime prevalence estimated to be as high as 18%; thus, they are also one of the most frequent psychiatric diseases in the general population [1].

Depressive and anxiety disorders have a complex etiology, involving both genetic and environmental factors. Heritability estimates range between 31% to 42% for major depressive disorder and between 20% to 40% for anxiety disorder [3, 4]. An important issue in the search for risk factors of anxiety and depressive disorders is the frequent co-morbidity between those phenotypes [5]. Depression and anxiety symptomatology are strongly linked [1, 6, 7], and the co-morbidity between those disorders results in lower social competence, in addition to more severe symptoms than those patients diagnosed with a single disorder [8].

One way to uncover additional information about the relationship between anxiety and depression is to examine the extent of shared genetic factors that influence these traits. Studies investigating the heritability of combined depression and anxiety disorders published to date have been based on twin samples mostly of European ancestry and, have shown a high genetic correlation between anxiety disorders and major depression [5]. An alternative approach that can aid in the identification of genetic susceptibility factors in those disorders is phenotypic variation. Depressive and anxiety disorders can be conceptualized as extreme diagnostic entities in a continuum of varying severity. Therefore, instead of relying on categorical phenotypes only, quantitative analysis of depressive and anxiety symptomatology can also improve the detection of meaningful genetic risk markers [9]. Indeed, heritability estimates were independently obtained from twin studies in the Netherlands and Australia showing that a common genetic factor is responsible for most of the variance in the phenotypic presentation of combined anxiety and depressive symptoms in the general population [10, 11].

The current study used the Hospital Anxiety Depression Scale (HADS) [12] to examine the heritability and shared genetic factors between symptoms of anxiety and depression from large extended families of a population that is highly admixed, mainly along the axis between European and African ancestry. The population chosen, the Baependi Heart Study cohort [13], is uniquely suited to our study purpose. Located in a rural town in Brazil (18,307 inhabitants recorded in the 2010 census), the study population is remarkably homogeneous in terms of their lifestyle, which remains very traditional. Thus, the ethnic background, living conditions, and family-based study design representing large and complex pedigrees, offer potential novel insights into the biology and genetic basis of depression and anxiety as well as other behavior traits [14].

## Materials and Methods

### Participants

This study included 1,375 individuals (57% female) belonging to extended pedigrees (95 families) aged 18–98 (mean ± SD = 42.52 ± 16.28) from the Baependi Heart Study, a genetic epidemiological study with a longitudinal design, who provided valid data to this study. The pedigrees had mean size of 24.15 ± 31.79 members and were from 3 to 4 generations in their majority (63%). There were 640 sibships with mean size of 2.45 ± 1.87 and the following numbers of main pairs of relatives were: parent/offspring– 3138; sibling/sibling– 2253; grandparent/grandchild– 2590; avuncular– 4418; half siblings– 40; and cousins: 3743. Further characteristics of the population and the sampling methodology have been described previously [13]. 6.4% (n = 88) of the participants were using antidepressant medication at the time of the interview. Subjects with cognitive impairments were not included. The authors assert that all procedures contributing to this work comply with the ethical standards of the relevant national and institutional committees on human experimentation and with the Helsinki Declaration of 1975, as revised in 2008. The study protocol was approved by Ethics committee of the Hospital das Clínicas, University of São Paulo, Brazil. Participation in the study was voluntary, and each volunteer provided informed written consent before participation. For illiterate participants or for those without the capacity to sign, the consent term was completely read by a researcher in the presence of an authorized representative of the volunteer, usually a relative, who signed the authorization according to the participant decision. Data were collected between December 2005 and January 2006.

### Instruments

Symptoms of depression and anxiety were assessed using a Portuguese translation of the HADS questionnaire made and validated for use in Brazil [15]. The scale consists of 14 self-administered items, with seven questions relating to anxiety and the other seven to depression [16]. The questions are based on a four-point Likert scale with scores ranging from 0 to 3. The Portuguese version of HADS has previously been shown to be easy to understand, and to have a high sensitivity and specificity [17]. The questionnaire was completed by the participants in the permanent field station of the Baependi project, and was read in its entirety by the researcher acting as a scribe to participants with an insufficient level of literacy.

### Statistical analysis

Comparisons among general characteristics were performed with SPSS 20 for Windows (IBM, Armonk, NY). Continuous variables like HADS scores were tested using ANOVA, and for dichotomous variables such as sex and age category differences, the chi-square test was used. The variance component model is a well-known tool for heritability estimates in family studies and was used to calculate polygenic heritability estimates [18]. In the most narrow sense, the heritability of a trait represents the proportion of the phenotypic variance attributable to addictive genetic effects and is given by h^2^ = σ^2^_a_/ σ^2^_p_, where σ^2^_a_ is the variance due to the addictive effects of genes, and σ^2^_p_ is the phenotypic variance. The overall phenotypic variance was estimated from the observed distribution of trait values in the sample, and was partitioned into genetic and environmental components using the observed covariance among family members, as Ω = 2ɸσ^2^_a_ + Iσ^2^_e_, where Ω is an “n x n” matrix of the “n” individuals in the data set, 2ɸ is the structuring matrix of the coefficient of relationship, and “I” is an identity matrix that represents the structuring matrix for σ^2^_e_, the variance due to residual environmental factors. Estimates of the mean and variance components were obtained using maximum likelihood methods [19, 20].

Joint analysis of multiple related phenotypes can be used to answer questions about the nature of the relationship between the traits and to increase power to localize genes influencing the traits [21, 22]. In this context, in the bivariate model, the genetic correlations (ρ_g_) and environmental correlations (ρ_e_) represent the additive polygenic effect of shared genes or common genetic effects on the two traits (pleiotropy) and environmental factors (non-genetic) in the phenotypic variance of each trait, respectively. The overall phenotypic correlation (ρ_p_) between two traits can be broken down into a genetic and environmental component:

$$\rho_{p}\;={\;\rho }_{\text{g}}\sqrt{h_{1}^{2}}\sqrt{h_{2}^{2}}+\rho_{\text{e}}\sqrt{1-h_{1}^{2}}\sqrt{1-h_{2}^{2}}$$ (1)

In this correlation, $h_{1}^{2}$ and $\;h_{2}^{2}$ are the heritabilities in trait 1 and trait 2. Using this nomenclature, the proportion of the total genetic variance that is due to shared genetic effects is estimated by the square of the genetic correlation (ρ_g_). Likelihood ratio tests were used to separately test the hypotheses that the two traits share no common genetic basis (H_0_:ρ_g_ = 0) and that the two traits have the identical genetic basis (H_0_:|ρ_g_| = 1).

In this study, the heritability of HADS-A and HADS-D as well as bivariate genetic correlations were conducted using the maximum likelihood estimate based variance components approach implemented in the statistical genetics software package, SOLAR [21]. Four models were fitted to the data: (1) unadjusted, (2) adjusted by age, (3) adjusted by gender and (4) adjusted by age and gender. In each adjusted model, the estimates of the parameters in question were calculated.

## Results

### Description of the sample

Socio-demographic characteristics of the sample are given in Table 1. All age categories were represented and there was no gross difference between genders. 62.9% of subjects were married or living with a partner. 41.6% of the sample had four years or less of schooling, and 86.4% of the sample had a monthly household income of less than R$1,500. No more than 1% of any used variable was missing.

**Table 1:** Socio-demographic characteristics of the sample.

	*Total*		*Males*		*Females*	
	*n*	*%*	*n*	*%*	*n*	*%*
Age category						
18–29 years	370	26.9	158	26.9	212	26.9
30–39 years	258	18.8	109	18.6	149	18.9
40–49 years	314	22.8	129	22.0	185	23.5
50–59 years	208	15.1	88	15.0	120	15.2
≥60 years	225	16.4	103	17.5	122	15.5
Marital status						
Married or living together	865	62.9	375	63.9	490	62.2
Single	368	26.8	178	30.3	190	24.1
Divorced	60	4.4	17	2.9	43	5.5
Widowed	73	5.3	13	2.2	60	7.6
Education						
≤ 4years	571	41.6	263	45.0	308	39.1
5–8 years	309	22.5	127	21.7	182	23.1
9–11 years	360	26.2	143	24.5	217	27.5
≥ 12 years	132	9.6	51	8.7	81	10.3
Household income ^a^						
Class E (Less than R$ 300.00)	249	18.7	100	17.5	149	19.6
Class D/C (R$ 300.00-R$ 1.500.00)	901	67.7	373	65.3	528	69.5
Class B (R$ 1.500.00-R$6.000.00)	172	12.9	93	16.3	79	10.4
Class A (More than R$ 6.000.00)	9	0.7	5	0.9	4	0.5

^a^ Monthly Income in Brazilian Real

### HADS values

The following coefficients of internal consistency (Cronbach’s alpha) were obtained: anxiety (α = 0.74), depression (α = 0.73), and total score (α = 0.83). Anxiety and depression were correlated with r = 0.59 (Pearson, p <0.0001) and r = 0.58 (Spearman's **ρ**, p<0.0001). Similar significant results were obtained when the sample was divided by gender: For males, r = 0.54, and for females, r = 0.59.

**Fig 1:**
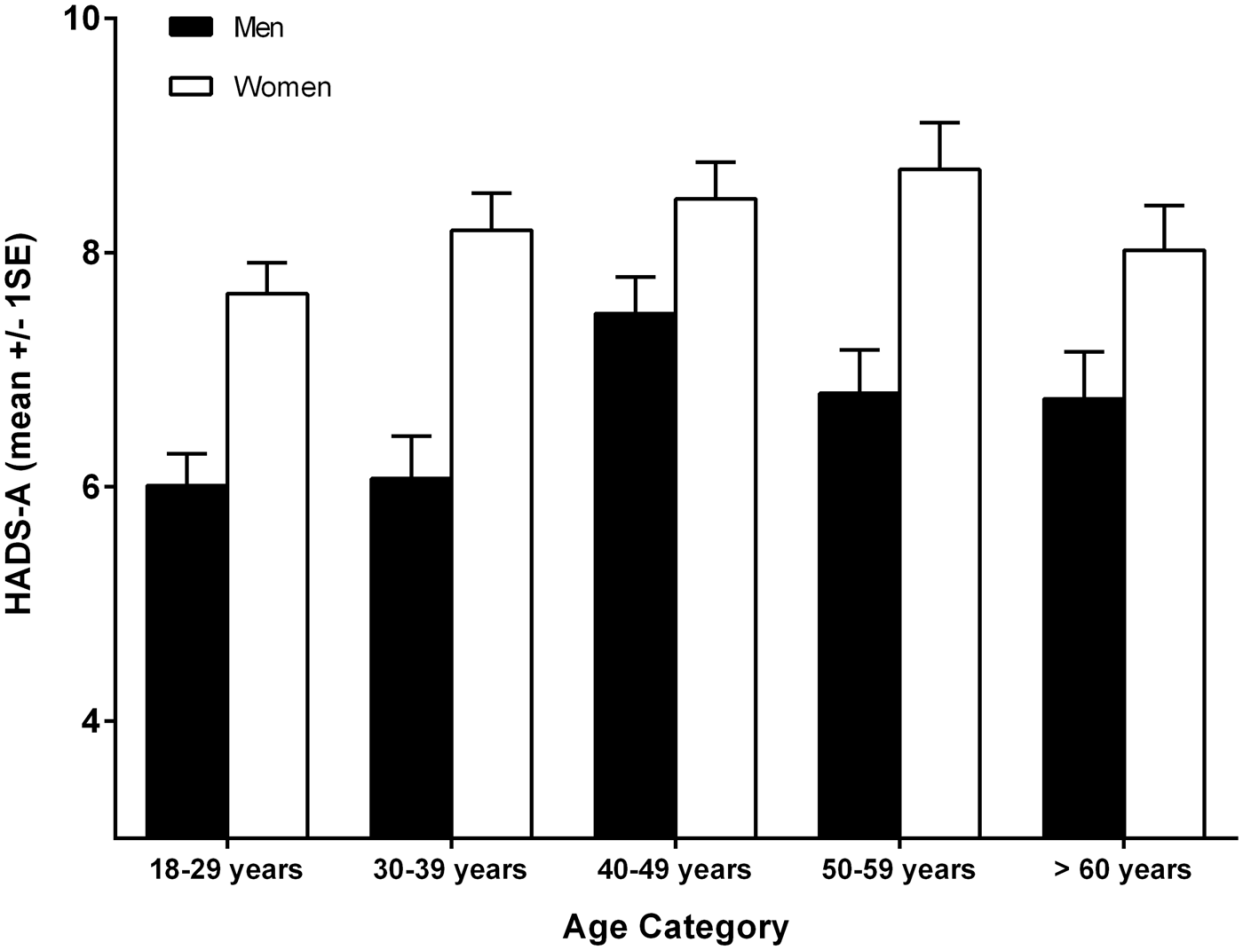
Mean HADS-A scores across age categories.

**Fig 2:**
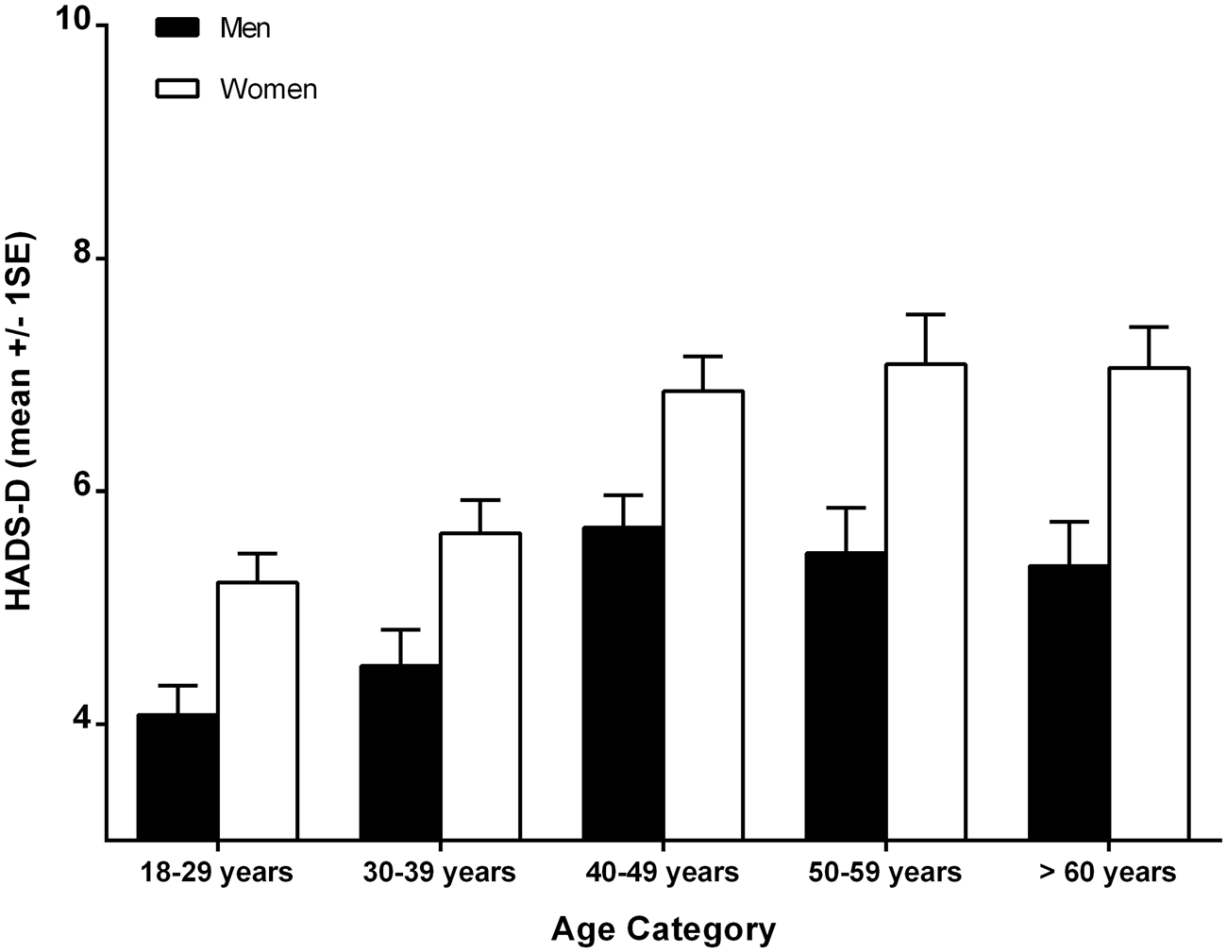
Mean HADS-D scores across age categories.

The mean scores and standard deviations in the HADS-A and HADS-D scale for the total population were 7.49±4.01 and 5.70±3.82, respectively, with responses ranging from 0 to 21. Women reported higher scores than men for anxiety symptoms (F = 53.32 p = 0.001). This gender difference persisted for depressive symptoms, but to a lesser degree (F = 40.57 p = 0.001). Age differences were noted in both anxiety (F = 4.02 p = 0.003) and depression (F = 13.19 p = 0.001). HADS-A and HADS-D mean scores increased with age until reaching their peak in the range of 40 to 59 years of age as illustrated in Figs 1 and 2, respectively.

### Heritability

Heritability estimates for HADS-A and HADS-D are shown in Table 2. Estimates were similar in the various adjusted models and were all statistically significant (p<0.0001). Heritability, without taking covariates into account (unadjusted model), was 0.28 for HADS-D and, 0.30 for HADS-A. When corrected for age and sex, the numbers increased to 0.30 (HADS-D) and 0.32 (HADS-A). The significant effects of age and sex for both phenotypes indicate that there is an increase in precision when these factors are accounted for. Thus, we also divided the sample into an older and a younger age group, the cut-off point being the 50% percentile (41 years), and ran a set of heritability estimates. A marked difference was found between these age groups. The younger age group had higher heritability estimate compared to the older age group for HADS-A (younger/older = 0.32/0.22) and, HADS-D scores (younger/older = 0.31/0.18). The added inclusion of age^2^ and age x gender did not change the results of heritability for any of the scores.

**Table 2:** Heritability estimates.

Model	h^2^_g_A	*p* values	h^2^_g_D	*p* values
**No adjustment**	0.30±0.06	<0.0001	0.28±0.06	<0.0001
**Gender**	0.31±0.06	<0.0001	0.29±0.06	<0.0001
**Age**	0.34±0.06	<0.0001	0.29±0.05	<0.0001
**Gender, age**	0.35±0.06	<0.0001	0.29±0.60	<0.0001
Gender*age	0.33±0.06	<0.0001	0.29±0.60	<0.0001

h^2^_g_ = heritability; A = anxiety scores; D = depression scores. Values are shown with standard deviation.

### Bivariate analyses

Testing the null hypothesis that the genetic correlation between HADS-A and HADS-D measures was zero (that is, that there is no shared genetic basis between HADS-A and HADS-D) resulted in highly significant P-values (< 10^−6^), and this hypothesis was therefore rejected (data not shown). A second null hypothesis that the genetic correlation between these traits is one (that is, the genetic bases HADS-A and HADS-D are identical) was also rejected as very unlikely (P<10^−4^) (data not shown).

The overall phenotypic correlation (ρ_p_) between HADS-A and HADS-D measures was broken down into a genetic (ρ_g_) and environment (ρ_e_) component according to Eq (1) described in the Methods section. The genetic correlation between HADS-A and HADS-D measures ranged from 0.78 to 0.81 through all models: the unadjusted, adjusted for gender, age, gender and age and interaction age *x* gender (Table 3). The decomposition of the phenotypic correlation revealed a stronger genetic than environmental correlation contribution to the overlap between depression and anxiety symptoms in all models.

**Table 3:** Bivariate analysis.

Model	ρ_p_	*p* values	ρ_g_	*p* values	ρ_e_	*p* values
**No adjustment**	0.59	<0.0001	0.78±0.07	<0.0001	0.52±0.04	<0.0001
**Gender**	0.58	<0.0001	0.79±0.04	<0.0001	0.50±0.04	<0.0001
**Age**	0.60	<0.0001	0.80±0.07	<0.0001	0.51±0.04	<0.0001
**Gender, age**	0.58	<0.0001	0.81±0.07	<0.0001	0.48±0.04	<0.0001
Gender*age	0.50	<0.0001	0.81±0.07	<0.0001	0.50±0.04	<0.0001

ρ_p_ = phenotypic correlation; ρ_g_ = genetic correlation; ρ_e_ = environmental correlation. Values are shown with standard deviation for ρ_g_ and ρ_e_.

The proportion of the total genetic variance due to shared genetic effects is estimated by the square of the genetic correlation, thus, in the best model, adjusted for gender and age, this proportion is 0.81^2^ = 0.66, suggesting that nearly 66% of the total genetic variance in HADS-A is shared with HADS-D.

## Discussion

In this study, we quantified the degree of shared genetic variance between anxiety and depression symptoms, assessed via the HADS-A and HADS-D sections, respectively, and also calculated estimates of heritability for the two sets of symptoms in a large sample of Brazilian families.

We found that approximately 66% of the genetic variance for HADS-A is shared with HADS-D, and vice versa. These results suggest that the same genetic factors influence the susceptibility to anxiety and depressive scores. A similar methodology was applied by Olvera and colleagues [23], who performed bivariate analyses in a family-based Mexican-American sample of shared genetic background between psychiatric disorders. That analysis found a strong genetic correlation in individuals concomitantly diagnosed with anxiety and depressive disorders (ρ_p_>0.75). Shared genetic correlations between anxiety and depressive symptoms have consistently been found in twin studies but those studies have all been based on cohorts of primarily European ancestry [10, 11]. Boomsma et al, 2000, in a study of Dutch adolescent and young adult twins, found a common genetic factor that influenced more than 40% of the variance explained by genetic factors for both depression and anxiety symptoms. Similarly, Kendler et al, 1987, found a common genetic factor that influenced the susceptibility of the concomitant presentation of depression and anxiety symptoms in an Australian adult twin cohort. Thus, our findings are unique in reporting a shared genetic background between the degree of anxiety and depression (as continuous variables) using the HADS scale in an admixed population, and confirmatory with respect to previous reports of a shared genetic background in comorbid symptoms seen in the general population in samples of mostly European ancestry.

The heritability estimates obtained in the present study were 0.30 HADS-D, and 0.32 for HADS-A. In the literature, heritability estimates for depression range from 0.17 to 0.78 and for anxiety from 0.25 to 0.60 [3, 24]. Heritability measures of anxiety and depression for this study lie on the lower side of the range of studies investigating both disorders [3, 24]. Differences in heritability estimates can be due to sample selection or experimental design. Those differences can be the result of age and/or gender-specific genetic effects that impact on those estimates. For example, we found in our very same cohort that individuals younger than 41 years old had a heritability estimate of 0.32 for anxiety compared to 0.22 in the oldest subset. This could also be the case in different samples with distinct age groups [25–27]. Despite methodological differences between classical twin design and extended pedigree design, rendering distinct heritability estimates, general results indicate that both are able to generate good estimates [28].

In order to discuss the relevance of our genetic findings, it is important to address particularities and possible limitations of the sample and study design. We found a lower frequency of symptoms of anxiety and depression in men compared with women, as reported in previous studies world-wide [29, 30]. Studies performed in Brazil showed the same tendency [15, 17], but were conducted in groups with health problems which tends to increase the HADS scores. It is interesting to note that both the depression and anxiety mean scores found in our population were higher than all the studies referred to above with exception to the Korean general population study where depression results (mean = 6.6) showed higher mean scores than in Baependi [31]. Possible explanations for higher scores in the Baependi population are the low level of education and income in this population (Table 1). These are important covariates to be considered, as low socio-economic status raises the odds for depression and anxiety and decreasing family income over time increased vulnerability to the symptoms of anxiety and depression [32, 33]. Educational level is one of the factors that comprises the socio-economic status index, and seems to exert a protective effect to anxiety and depression disorders. In an 11-year follow up study, people with a low educational level (often resulting in professions involving hard manual work) moved towards higher anxiety and depression scores when compared to others with high level of schooling [34].

The inclusivity of our study, a distinguishing aspect of this study, could also be a reason for the scores being higher than in other general population studies. All participants with valid scores were included, independent of age (participants were aged up to 98), sex, use of medication, socio-economic status, and health condition. The subjectivity of self-assessment scales like HADS, although extensively used in epidemiological studies, can be a potential source of bias as well [26]. Ultimately, given the unique and in many ways advantageous features of this study design (a highly admixed population living in a rural town with a very conservative lifestyle), and some unique complications (e.g. a high level of illiteracy), it becomes difficult to compare it with previous findings.

## Conclusions

The finding of a high proportion of shared genetic factors between continuous measures of depression and anxiety shows that the well-known clinical correlation between these systems is reflected on a profound biological level. Furthermore, evidence for shared genetic effects has promising implications for future molecular genetics studies, because they may increase power to localize genes influencing these traits in genome-wide association studies [35].

## References

[1] Kessler RC, Chiu WT, Demler O, Merikangas KR, Walters EE. Prevalence, severity, and comorbidity of 12-month DSM-IV disorders in the National Comorbidity Survey Replication. Arch Gen Psychiatry. 2005;62(6):617–27. 10.1001/archpsyc.62.6.617 15939839 PMC2847357

[2] Kessler RC. The global burden of anxiety and mood disorders: putting the European Study of the Epidemiology of Mental Disorders (ESEMeD) findings into perspective. J Clin Psychiatry. 2007;68 Suppl 2:10–9. 17288502 PMC1852440

[3] Sullivan PF, Neale MC, Kendler KS. Genetic epidemiology of major depression: review and meta-analysis. Am J Psychiatry. 2000;157(10):1552–62. . 11007705 10.1176/appi.ajp.157.10.1552

[4] Smoller JW, Block SR, Young MM. Genetics of anxiety disorders: the complex road from DSM to DNA. Depress Anxiety. 2009;26(11):965–75. 10.1002/da.20623 . 19885930

[5] Middeldorp CM, Cath DC, Van Dyck R, Boomsma DI. The co-morbidity of anxiety and depression in the perspective of genetic epidemiology. A review of twin and family studies. Psychol Med. 2005;35(5):611–24. . 15918338 10.1017/s003329170400412x

[6] Zbozinek TD, Rose RD, Wolitzky-Taylor KB, Sherbourne C, Sullivan G, Stein MB, et al Diagnostic overlap of generalized anxiety disorder and major depressive disorder in a primary care sample. Depress Anxiety. 2012;29(12):1065–71. 10.1002/da.22026 23184657 PMC3629816

[7] Mathew AR, Pettit JW, Lewinsohn PM, Seeley JR, Roberts RE. Co-morbidity between major depressive disorder and anxiety disorders: shared etiology or direct causation? Psychol Med. 2011;41(10):2023–34. 10.1017/S0033291711000407 21439108 PMC3713851

[8] Cerdá M, Sagdeo A, Johnson J, Galea S. Genetic and environmental influences on psychiatric comorbidity: a systematic review. J Affect Disord. 2010;126(1–2):14–38. 10.1016/j.jad.2009.11.006 20004978 PMC2888715

[9] Flint J, Kendler KS. The genetics of major depression. Neuron. 2014;81(3):484–503. 10.1016/j.neuron.2014.01.027 24507187 PMC3919201

[10] Kendler KS, Heath AC, Martin NG, Eaves LJ. Symptoms of anxiety and symptoms of depression. Same genes, different environments? Arch Gen Psychiatry. 1987;44(5):451–7. . 3579496 10.1001/archpsyc.1987.01800170073010

[11] Boomsma DI, Beem AL, van den Berg M, Dolan CV, Koopmans JR, Vink JM, et al Netherlands twin family study of anxious depression (NETSAD). Twin Res. 2000;3(4):323–34. . 11463154 10.1375/136905200320565300

[12] Crawford JR, Henry JD. The Depression Anxiety Stress Scales (DASS): normative data and latent structure in a large non-clinical sample. Br J Clin Psychol. 2003;42(Pt 2):111–31. 10.1348/014466503321903544 . 12828802

[13] de Oliveira CM, Pereira AC, de Andrade M, Soler JM, Krieger JE. Heritability of cardiovascular risk factors in a Brazilian population: Baependi Heart Study. BMC Med Genet. 2008;9:32 10.1186/1471-2350-9-32 18430212 PMC2386446

[14] von Schantz M, Taporoski TP, Horimoto AR, Duarte NE, Vallada H, Krieger JE, et al Distribution and heritability of diurnal preference (chronotype) in a rural Brazilian family-based cohort, the Baependi study. Sci Rep. 2015;5:9214 10.1038/srep09214 25782397 PMC4363835

[15] Botega NJ, Bio MR, Zomignani MA, Garcia C, Pereira WA. [Mood disorders among inpatients in ambulatory and validation of the anxiety and depression scale HAD]. Rev Saude Publica. 1995;29(5):355–63. . 8731275 10.1590/s0034-89101995000500004

[16] Zigmond AS, Snaith RP. The hospital anxiety and depression scale. Acta Psychiatr Scand. 1983;67(6):361–70. . 6880820 10.1111/j.1600-0447.1983.tb09716.x

[17] Soares-Filho GL, Freire RC, Biancha K, Pacheco T, Volschan A, Valença AM, et al Use of the hospital anxiety and depression scale (HADS) in a cardiac emergency room: chest pain unit. Clinics (Sao Paulo). 2009;64(3):209–14. 19330247 10.1590/S1807-59322009000300011 PMC2666460

[18] Thompson EA. Identity by descent: variation in meiosis, across genomes, and in populations. Genetics. 2013;194(2):301–26. 10.1534/genetics.112.148825 23733848 PMC3664843

[19] de Andrade M, Amos CI, Thiel TJ. Methods to estimate genetic components of variance for quantitative traits in family studies. Genet Epidemiol. 1999;17(1):64–76. 10.1002/(SICI)1098-2272(1999)17:1<64::AID-GEPI5>3.0.CO;2-M . 10323185

[20] Almasy L, Blangero J. Variance component methods for analysis of complex phenotypes. Cold Spring Harb Protoc. 2010;2010(5):pdb.top77 10.1101/pdb.top77 20439422 PMC3064490

[21] Almasy L, Blangero J. Multipoint quantitative-trait linkage analysis in general pedigrees. Am J Hum Genet. 1998;62(5):1198–211. 10.1086/301844 9545414 PMC1377101

[22] Lange K, Boehnke M. Extensions to pedigree analysis. IV. Covariance components models for multivariate traits. Am J Med Genet. 1983;14(3):513–24. 10.1002/ajmg.1320140315 . 6859102

[23] Olvera RL, Bearden CE, Velligan DI, Almasy L, Carless MA, Curran JE, et al Common genetic influences on depression, alcohol, and substance use disorders in Mexican-American families. Am J Med Genet B Neuropsychiatr Genet. 2011;156B(5):561–8. 10.1002/ajmg.b.31196 21557468 PMC3112290

[24] McGrath LM, Weill S, Robinson EB, Macrae R, Smoller JW. Bringing a developmental perspective to anxiety genetics. Dev Psychopathol. 2012;24(4):1179–93. 10.1017/S0954579412000636 23062290 PMC3721501

[25] Czajkowski N, Røysamb E, Reichborn-Kjennerud T, Tambs K. A population based family study of symptoms of anxiety and depression: the HUNT study. J Affect Disord. 2010;125(1–3):355–60. 10.1016/j.jad.2010.01.006 . 20494447

[26] López-León S, Aulchenko YS, Tiemeier H, Oostra BA, van Duijn CM, Janssens AC. Shared genetic factors in the co-occurrence of symptoms of depression and cardiovascular risk factors. J Affect Disord. 2010;122(3):247–52. 10.1016/j.jad.2009.07.008 . 19674795

[27] Tambs K. Transmission of symptoms of anxiety and depression in nuclear families. J Affect Disord. 1991;21(2):117–26. . 1827639 10.1016/0165-0327(91)90058-z

[28] Docherty AR, Kremen WS, Panizzon MS, Prom-Wormley EC, Franz CE, Lyons MJ, et al Comparison of Twin and Extended Pedigree Designs for Obtaining Heritability Estimates. Behav Genet. 2015;45(4):461–6. 10.1007/s10519-015-9720-z 25894926 PMC4459911

[29] Crawford JR, Henry JD, Crombie C, Taylor EP. Normative data for the HADS from a large non-clinical sample. Br J Clin Psychol. 2001;40(Pt 4):429–34. . 11760618 10.1348/014466501163904

[30] Hinz A, Brähler E. Normative values for the hospital anxiety and depression scale (HADS) in the general German population. J Psychosom Res. 2011;71(2):74–8. 10.1016/j.jpsychores.2011.01.005 . 21767686

[31] Yun YH, Kim SH, Lee KM, Park SM, Kim YM. Age, sex, and comorbidities were considered in comparing reference data for health-related quality of life in the general and cancer populations. J Clin Epidemiol. 2007;60(11):1164–75. 10.1016/j.jclinepi.2006.12.014 . 17938059

[32] Melchior M, Chastang JF, Walburg V, Arseneault L, Galéra C, Fombonne E. Family income and youths' symptoms of depression and anxiety: a longitudinal study of the French GAZEL Youth cohort. Depress Anxiety. 2010;27(12):1095–103. 10.1002/da.20761 . 21132845

[33] Lorant V, Deliège D, Eaton W, Robert A, Philippot P, Ansseau M. Socioeconomic inequalities in depression: a meta-analysis. Am J Epidemiol. 2003;157(2):98–112. . 12522017 10.1093/aje/kwf182

[34] Bjelland I, Krokstad S, Mykletun A, Dahl AA, Tell GS, Tambs K. Does a higher educational level protect against anxiety and depression? The HUNT study. Soc Sci Med. 2008;66(6):1334–45. 10.1016/j.socscimed.2007.12.019 . 18234406

[35] Lee SH, Ripke S, Neale BM, Faraone SV, Purcell SM, Perlis RH, et al Genetic relationship between five psychiatric disorders estimated from genome-wide SNPs. Nat Genet. 2013;45(9):984–94. 10.1038/ng.2711 23933821 PMC3800159

